# Gold Nanoparticles in Biomedical Applications: Synthesis, Functionalization, and Recent Advances

**DOI:** 10.3390/molecules31010017

**Published:** 2025-12-20

**Authors:** Massa Zahdeh, Rafik Karaman

**Affiliations:** 1 Pharmaceutical Sciences Department, Faculty of Pharmacy, Al-Quds University, Jerusalem 20002, Palestine; zahdehmassa@gmail.com; 2 Department of Sciences, University of Basilicata, Via dell’Ateneo Lucano 10, 85100 Potenza, Italy

**Keywords:** gold nanoparticles, AuNP synthesis, functionalization, green synthesis, nanomedicine, advanced nanotechnology

## Abstract

Background: Gold nanoparticles (AuNPs) are metallic nanoparticles with strong biomedical potential and have FDA approval. Their nanoscale size, optical tunability, and biocompatibility allow them to be used for tumor-targeted delivery, photothermal therapy, imaging contrast, radiosensitization, gene transfection, biosensing, personalized medicine and AI-supported healthcare solutions. These properties made AuNPs a game-changing tool in nanomedicine. Methods: Google Scholar, PubMed, Scopus and ScienceDirect were used to search the literature with keywords related to gold nanoparticles, synthesis, functionalization and advanced applications in biomedicine. The search mainly focused on studies published between 2018–2025, and older landmark papers were only included when needed to describe classical synthesis. Results: Standard AuNP synthesis and functionalization approaches were compared with advanced methods such as green synthesis, microfluidic synthesis, polymer functionalization and AI-supported synthesis optimization. AuNPs moved from traditional drug administration and basic diagnostics into multiplex imaging, targeted therapy, hybrid theranostics, spectral CT imaging, gene delivery and CRISPR-related applications. Conclusions: This review demonstrates the evolution of AuNPs in biomedicine from traditional nanoparticles to sophisticated multifunctional nanosystems. To the best of our knowledge, this is the first assessment that explicitly contrasts sophisticated AuNP techniques with conventional procedures in biomedical applications.

## 1. Introduction

In nanotechnology, materials are designed and used by manipulating their size and shape in the nanoscale (1–100 nm) range [1]. The nanomaterials known as nanoparticles (NPs) are important in many fields, such as chemistry, biology, physics, medicine, and sensing, because of their special qualities and smaller sizes [2,3]. Given their similar scale to biological molecules, nanoparticles (NPs) can be designed to carry out a variety of tasks in medical settings. This relates to the subject of nanomedicine, which uses the physical and chemical properties of NPs to diagnose and cure diseases at the molecular level [4]. Noble metals including copper (Cu), mercury (Hg), silver (Ag), platinum (Pt), and gold (Au) can produce a variety of nanoparticles because of their remarkable adaptability to a wide range of uses [5]. A variety of shapes and architectures, including nanospheres, nanobranches, nanobipyramids, nanorods, nanocubes, nanoshells, nanowires, and nanocages, each created for a particular use, make AuNPs unique [6]. Although traditional AuNP synthesis techniques, such as chemical and physical methods, are still vital and the foundation for AuNP production, advanced synthesis techniques, such as microfluidics, green eco-friendly systems, and machine learning (ML)-integrated nanoparticles, are finding their way into cutting-edge biomedical applications [7,8,9]. To discuss how sophisticated synthesis techniques contribute to the development of biomedical applications and insights into precision medicine, a comparative study of different approaches is required.

They are widely used in fields such as therapy and diagnostics due to their remarkable optical, physiochemical, and biocompatible qualities, including surface plasmon resonance (SPR), surface-enhanced Raman spectroscopy (SERS), and computational tomography (CT), as well as functionalizing ligands and polymers [2]. The innate immune system of the body does not react to AuNPs as a foreign particle; hence, they are regarded as safe nanoparticles. As demonstrated by many investigations, AuNPs are not cytotoxic and might not be in charge of the release of proinflammatory cytokines such as TNF-α and IL1-β [10,11].

In addition, AuNPs are ideal contrast agents for safer and more targeted radiological diagnostics and treatments because of their SPN behavior, low toxicity, and biocompatibility when compared to other NPs [12]. They are also appropriate particles for delivery systems, such as medications, genes, and proteins. Furthermore, AuNPs are being studied increasingly in advanced biomedical and bioengineering applications such as regenerative medicine, immunological modulation, implants, gene editing such as CRISPR-associated protein 9 studies, and cancer theranostics [13,14].

A number of thorough reviews have summarized various aspects of gold nanoparticles in biomedicine in recent years. An extensive review of AuNP production, surface modification, and medical applications was given by Hu et al. [15]. Ferreira et al. concentrated on AuNPs as vectors for nucleic acid delivery in cancer therapies [13], Luo et al. examined their function as contrast agents for cancer imaging [16], and Anik et al. examined AuNPs in biological and clinical contexts with a focus on general features [2]. Although these reviews cover significant aspects of AuNP research, they often tackle biological performance, synthesis pathways, and functionalization strategies independently, lacking an integrated evaluation of established and cutting-edge synthesis technologies within a single framework.

The majority of reviews that are currently available either concentrate on AuNP synthesis and general physico-chemical properties or on particular biomedical applications, despite the fact that AuNPs have been extensively studied in both conventional and cutting-edge biological domains. However, they do not offer an integrated comparison that connects synthesis strategies with surface functionalization and advanced biomedical performance. In this review, we compare conventional, green, and microfluidic AuNP synthesis techniques in a methodical manner, talk about functionalization techniques and how they affect pharmacological behavior and colloidal stability, and map these cutting-edge design strategies onto existing and new biomedical applications.

## 2. Materials and Methods

Common databases including Google Scholar, PubMed, Scopus, and ScienceDirect were used to conduct the literature search. Boolean operators (AND, OR) were used in conjunction with pertinent keywords, such as “gold nanoparticles”, “synthesis”, “advancements”, “therapy”, “diagnostics”, “personalized medicine”, and “artificial intelligence”, to ensure that pertinent research were included. Full-text English-language publications that described the synthesis, characteristics, or therapeutic/diagnostic uses of gold nanoparticles were the main focus of this search. For this review, I mainly focused on studies published between 2018 and 2025. Older landmark papers were only used when needed to reference foundational synthesis principles. To enhance the review’s quality, other filters were used, such as restricting it to peer-reviewed journal articles and those pertaining to medicine, also to minimize the risk of missing influential contributions, reference lists of selected key publications were also checked alongside database searching. Articles with an environmental purpose or unrelated to gold nanoparticles were carefully eliminated. The selection of pertinent literature for the gold nanoparticle study was guaranteed by this methodological approach.

## 3. Characteristics and Properties of AuNPs

AuNPs have a number of properties that make them suitable for use in medicine, including antibacterial agents, anticancer agents, tissue engineering, drug transport, cellular probes, photomedicine, and biosensing [17]. The features of AuNPs that make them appropriate for biomedical applications, however, will be the main topic of this section. Because of their size, shape, surface characteristics, and optical qualities, AuNPs are promising drug carriers [18].

### 3.1. Shape and Size

**Figure 1:**
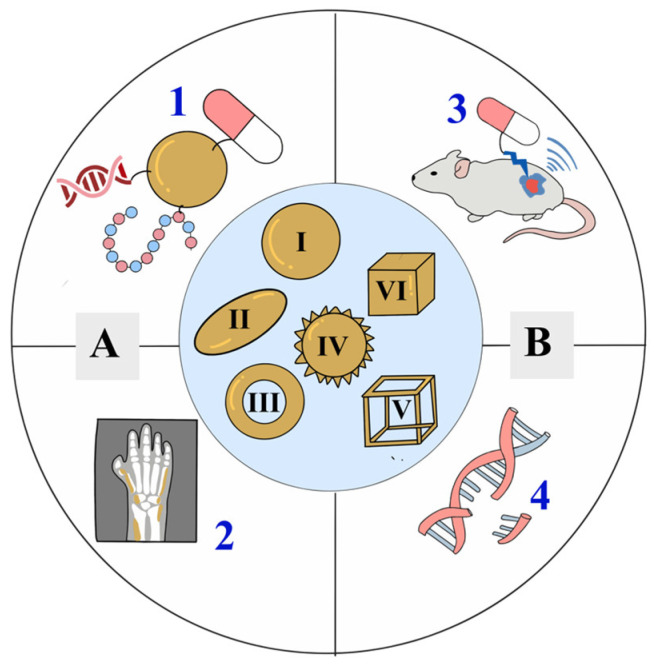
Gold Nanoparticle biomedical applications and morphologies: A represents conventional applications including (1) Molecular Delivery Systems and (2) Basic Diagnostics and Imaging, while B represents advanced applications such as (3) Theranostics and (4) Advanced Gene Editing. Nanoparticle shapes shown are I: Nanosphere, II: Nanorod, III: Nanoshell, IV: Nanoflower, V: Nanocage, and VI: Nanocube.

AuNPs have unique properties in biological sensing and detection due to their unique size and shape. Figure 1 shows the many forms of AuNP. Every shape of AuNPs, including branching, nanospheres, nanorods, nanoshells, nanocubes, nanocages, and more, has a particular use [19,20]. Because it directly influences AuNPs’ optical and catalytic function, this structural diversity is important. Changing the particle shape can change the localized surface plasmon resonance (LSPR) frequency and expand or narrow the optical absorption band, which directly affects light absorption, photothermal conversion efficiency, and sensing performance [21,22]. For example, anisotropic forms such as nanorods and nanocages have more plasmon modes and greater resonance in specific wavelength regions than spherical particles, which makes them more suitable for photothermal and imaging applications [23].

For example, the catalytic performance of various AuNP forms for the reduction of p-nitroaniline was investigated by Jiji et al. In comparison to their spherical counterparts, the results showed that AuNPs nanorods had a higher catalytic efficiency [24]. Moreover, spherical AuNPs are more frequently employed in biological detection than other types due to their well-known characteristics, which include low toxicity, a high surface-to-volume ratio, and biocompatibility [20]. With an average diameter ranging from 1 nm to 100 nm [25], AuNPs exhibit size-dependent properties that dictate their use in different applications. Moreover, Mirkin et al. were among the first to show that gold nanoparticles could be used for biosensing through a DNA-guided assembly method based on 13 nm spherical AuNPs, where the aggregation process could be reversed by heating. This work helped establish 13 nm AuNPs as the most stable and commonly used platform for colorimetric biosensing [26].

Shafiqa et al. demonstrated that AuNPs with sizes between 20 and 100 nm have higher absorption efficiency as they get bigger, peaking at 70 nm [27]. Using AuNPs with sizes ranging from 6 to 22 nm, Suchomel et al. conducted another investigation on catalytic behavior and found that catalytic activity increases with decreasing size, primarily due to a larger surface-area-to-volume ratio [28].

### 3.2. Surface Characteristics

The biodistribution, stability, and interaction of AuNPs are largely determined by their surface properties, since positive-charged AuNPs interact with cells more readily than negative or neutral ones [29]. In order to effectively transfer plasmid DNA (pVAXmIL-2) into C2C12 cells, Noh et al. evaluated cationic AuNPs. The results demonstrated that this surface characteristic is essential for AuNPs to bond well with negatively charged cell membranes [30]. However, research has shown that AuNPs with a negative charge are less harmful. The toxicity of AuNPs to the small freshwater crustacean Daphnia Magna was investigated by Bozich et al., who concentrated on surface charge. Positively charged AuNPs were shown to be more hazardous than negative ones [31].

### 3.3. Optical Properties

**Figure 2:**
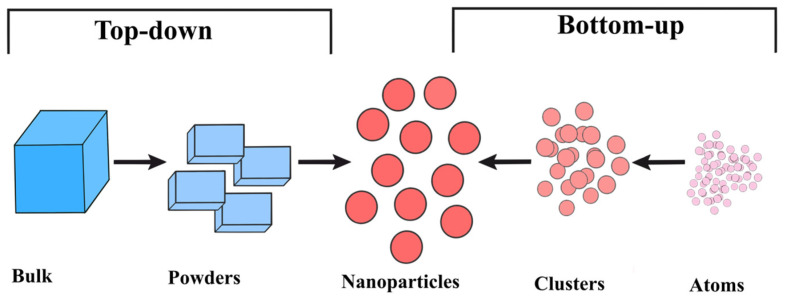
The top-down and bottom-up approaches for AuNP synthesis.

AuNPs’ optical characteristics are largely determined by their localized surface plasmon resonance (LSPR), a phenomenon where light causes the conduction electrons on the nanoparticle’s surface to oscillate collectively. This allows AuNPs to absorb and scatter light for a variety of uses, such as labeling, imaging, and sensing (see Figure 2 [32]). Amendola et al. provided a detailed explanation of the fundamental ideas behind surface plasmon resonance in gold nanoparticles. They showed that the optical properties of the surrounding medium and the separation between neighboring particles control the plasmonic response of AuNPs in addition to particle size and shape. The plasmon band usually shifts toward longer wavelengths and widens as particle size increases due to radiation losses and phase retardation effects. Variations in particle shape offer novel resonance modes and expand the range of possible optical responses. Additionally, aggregation and tight particle–particle interactions greatly modify absorption and scattering behavior through plasmon coupling [33]. In bioimaging studies, for example, AuNPs are widely utilized for tumor identification in oncology because they improve contrast and resolution in a biological context. They are also used in highly sensitive biosensors because of their LSPR effects [16,34]. Jin-Ho Lee et al. described how AuNPs function as effective colorimetric sensing elements through LSPR. They explained that changes in the surrounding environment or nanoparticle aggregation produce visible color shifts that can be observed with the naked eye. They also summarized a wide range of AuNP-based colorimetric platforms developed for detecting DNA, proteins, enzymes, and cancer biomarkers [35]. Furthermore, LSPR and other optical processes are influenced by the morphological characteristics of AuNPs. For example, in contrast to other basic AuNP shapes, branch structures such as nanostars exhibit several shape resonance peaks because of their asymmetric shapes that hold distinct optical trains [21].

AuNPs also interact with other optical processes, such as Förster resonance energy transfer (FRET), which inhibits fluorescence by quenching the surface plasmon band fluorescence of a fluorophore and then absorbing photons through a photo-induced electron transfer [20]. AuNPs are useful for the development of biosensors because of their quenching characteristic. Wang et al. reported the successful use of AuNP-based electrochemical sensors for ultra-low-level detection of glucose and prostate-specific antigen (PSA), colorimetric assays based on aggregation-induced plasmon shifts for visual diagnosis, and LSPR platforms for the rapid detection of bacterial and viral pathogens, demonstrating the clinical and diagnostic power of AuNP-enabled biosensing [36].

Furthermore, the exceptional photothermal and photodynamic characteristics of AuNPs are essential for oncology [37]. Under certain circumstances, photothermal therapy (PTT) kills cells by converting the energy of absorbed light into heat [38]. Faid et al. exhibited citrate-capped AuNPs as photothermal agents in tests on the Michigan Cancer Foundation-7 (MCF-7) breast cancer cell line and discovered that photothermal efficiency is higher than irradiation with a 532 nm laser alone. While near-infrared (NIR) light between 700 and 900 nm is usually employed for photothermal therapy in deeper tissues, where tissue penetration is significantly stronger, this wavelength is used for laboratory experiments [39]. Gold nanospheres exhibit a plasmon resonance in the visible range (around 520–540 nm), however elongated particles like gold nanorods can push the plasmon band into the NIR region, making them more suitable for in-vivo photothermal applications. Under suitable irradiation conditions, photothermal efficiency is increased by both AuNP concentration and NIR absorption strength [33,37]. Furthermore, photodynamic therapy (PDT) reduces cancer cells by activating and generating ROS to kill cancerous cells by combining a photosensitizing drug with molecular oxygen [22]. The fundamental characteristics of AuNPs are used to highlight their impact on novel biological applications and to contrast traditional and cutting-edge production and functionalization techniques.

## 4. Synthesis of Gold Nanoparticles

**Figure 3:**
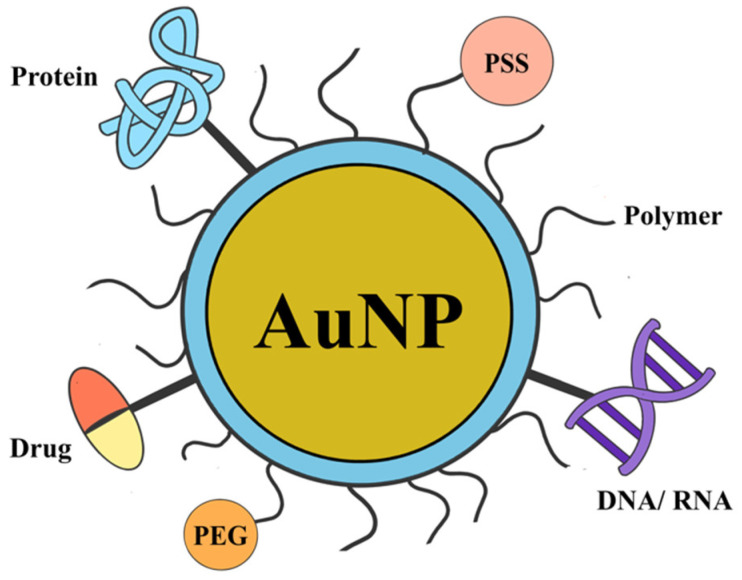
Functionalization of gold nanoparticles through conventional ligand attachment and advanced polymer coating to enhance stability and biomedical use.

Like many other types of inorganic nanomaterials, AuNPs can be synthesized using a variety of methods that fall into two primary categories: “top-down” and “bottom-up” ways, as illustrated in Figure 3 [40,41,42].

The top-down method involves using a variety of techniques to break down bulk materials into nanoparticles. The bottom-up method, on the other hand, starts at the atomic level and progresses to nanoparticles with the necessary sizes and shapes [40]. Additionally, AuNPs can be produced using a variety of techniques, such as chemical, physical, biological, and green synthesis, each of which has unique properties, as Table 1 [42] illustrates.

### 4.1. Top-Down vs. Bottom-Up Approaches

The top-down method is a subtractive procedure that involves breaking down large materials into smaller ones. In order to transform bulk gold into AuNP, this method usually uses physical processes such as laser ablation, aerosol technology, UV and IR irradiation, and ion sputtering. The top-down method, however, is severely limited in its ability to regulate the AuNPs’ surface and structure, which greatly affects their chemical and physical characteristics. During these synthetic processes, a great deal of energy is needed to sustain high temperature and pressure conditions, and size distribution is also unpredictable. Thus, it is exceedingly uneconomical and difficult to achieve product criteria [40,41]. Using chemical agents that will develop into nanoscale clusters and particles, the bottom-up technique creates nanoparticles from the atomic level [43]. The first step involves reducing a gold precursor, usually a salt, in an aqueous solution. The size of the nanoparticles is directly impacted by the reducing agent used, which can range from sodium borohydride to citrate. The second is the stabilization of AuNPs, which is accomplished by electrostatic stabilization or covalent bonding formed by coatings. The intended use and how the coating interacts with the target determine which coating is best. It regulates AuNP characteristics, as opposed to the top-down method. Its optimization, however, can assist in handling difficulties brought on by the synthesis variables [41].

### 4.2. Conventional Methods of Synthesis

AuNPs can be synthesized using two different methods: (1) physical methods including laser ablation, sputter deposition, ion implantation, γ-irradiation, optical lithography, microwave (MW), ultrasound (US), ultraviolet (UV), and sputter deposition; (2) chemical reduction of metal ions in solutions through the use of stabilizing agents and chemical agents like sodium hydroxide (NaOH), sodium borohydride (NaBH4), cetyl-trimethylammonium bromide (CTAB), lithium aluminum hydride (LiAlH4), sodium dodecyl sulfate (SDS), ethylene glycol (EG), and sodium citrate. The following sections will go into greater detail on these syntheses.

#### 4.2.1. Chemical Methods

The chemical reduction of metal ions in solutions is the most widely used technique for creating AuNPs. These techniques mostly use stabilizing and reducing chemicals to reduce gold ions Au(III) into gold atoms Au(0). To regulate the ultimate size and form of AuNPs, this technique also relies on temperature, pH, the solvent utilized, and the reducing/stabilizing agent. Numerous chemical techniques have developed over time, each with specific uses and needs. These include the Seed-Mediated Growth, Brust, and Turkevich approaches [37].

A popular technique that produces 10–100 nm hydrophilic spherical AuNPS, the Turkevich method is the first chemical synthesis of AuNPs. It uses reducing agents such as amino acids, ascorbic acid, UV light, or citrate to convert gold ions (Au^3+^) to gold atoms (Au^0^), and it also requires stabilization with various stabilizing agents [44]. Due to the narrow range of AuNPs, the method’s first uses were constrained. Advances in the fundamental technique, however, have allowed researchers to increase the size range by altering the proportion of stabilizing and reducing chemicals, resulting in AuNPs with diameters ranging from 16 to 147 nm [45].

A two-phase reaction employing organic solvents, the Brust–Schiffrin approach was initially described in 1994 and produces AuNPs with a size range of 1.5–5.2 nm [46]. In order to move gold salt from its aqueous solution (such as toluene) to an organic solvent, the technique uses a phase transfer such as tetraoctylammonium bromide. Sodium borohydride is also used as a reducing agent, and alkanethiols are used for stabilization [45].

More recently, it has been thought that Seed-Mediated Growth synthesis provides very accurate and efficient control over the size and form of AuNPs. Only spherical AuNPs can be produced using the first two techniques. However, the creation of nanometric gold rods has been the main use for this technique. In order to stop further nucleation and accelerate the synthesis of AuNPs with a rod shape, this method first synthesizes seed particles by reducing gold salt in the presence of NaBH4 and then transferring the seed particles to a metal salt and a weak reducing agent, such as ascorbic acid [45,47].

#### 4.2.2. Physical Methods

The top-down strategies used in physical procedures for AuNP production are predicated on breaking down bulk materials into tiny particles. These techniques rely on energy-intensive procedures, primarily physical ion implantation techniques and laser ablation.

In laser ablation, AuNPs are deposited on a surface using the sputtering approach when a metal-based material is utilized. The first step in this process is the target’s exposure to high-energy inert gas ions [48]. In aqueous solution, laser ablation also modifies AuNPs’ surface area, geometric shape, characteristics, fragmentation, and aggregation. According to one study by Vinod et al. (2017) [49], When stimulated by irradiation from a 532 nm laser, these particles exhibit photothermal activity [47]. However, Riedel et al. (2020) used enhanced pulsed laser ablation in liquid (PLAL) to create spherical, silica-coated AuNPs, which represents a significant advancement in the large-scale manufacturing of nanoparticles [50].

Ion implantation, which has been widely employed to create AuNPs with precise physical, chemical, and biological properties, is another potential technique for AuNP synthesis. Nie et al. (2018) [51] demonstrated the use of ion implantation followed by thermal annealing to create embedded AuNPs in Nd as YAG single crystals. The Nd: YAG crystals’ linear and non-linear absorption has been greatly increased [51].

### 4.3. Advanced Methods of Synthesis

The necessity for effective synthesis techniques has grown dramatically as AuNP applications continue to grow. In order to produce extremely stable, secure, and sophisticated AuNPs for a variety of biomedical uses, researchers are becoming interested in developing novel, multimodal pathways. Various alternative ways have been developed to synthesize biocompatible AuNPs for novel applications in order to address the difficulties associated with the traditional chemical and physical production methods.

#### 4.3.1. Green Synthesis

Green synthesis, which uses natural bioresources like plants and microorganisms to produce different AuNPs without the use of hazardous chemicals, is crucial because it improves biocompatibility, lowers costs, and has a less environmental impact [7,52]. Plant materials such as leaves, fruits, bark, flowers, peels, seeds, rhizome, and roots are used in a single step to biosynthesize AuNPs. These parts are cleaned with distilled water, dried, chopped into small pieces, and then cooked to a particular temperature. The extract, which contains secondary metabolites that serve as stabilizing and reducing agents for the effective synthesis of AuNPs, is next purified using filtration procedures. In addition to being easy to scale up, the entire process is environmentally beneficial. Although reports of the synthesis of AuNPs from other plant components have been made, leaves are often utilized [53]. As an illustration, Farihah et al. demonstrated that AuNPs could be produced utilizing HAuCl_4_ and red shoot leaf extract as a bioreactor, and they assessed the antioxidant activity of these particles. Comparing AuNPs’ antioxidant efficacy to other synthesis methods, the results demonstrated that environmentally friendly green synthesis significantly affects it [54]. Utilizing microbial synthesis in conjunction with computational modeling, further biogenic investigations have demonstrated optimal size control and reproducibility of AuNPs [55]. Another study by Islam et al. used the leaf extract of the plant *Salix alba* L., which is a member of the Salicaceae family, as a precursor in the formation of aspirin, highlighting the significance of leaves in green synthesis. Highlighting the benefits of green synthesis in medicinal applications, the leaf extract of *S. alba* decreased aqueous gold ions, resulting in the effective synthesis of AuNPs with antifungal activity, pain-relieving, and muscle-relaxing properties [56].

While green synthesis is attractive for its environmental and biological benefits, reproducibility and batch-to-batch consistency remain significant challenges. Iravani pointed out that the chemical makeup of plant extracts can differ with the plant species, cultivation conditions, season, and extraction procedure, and these variations directly affect the size, shape, and stability of the produced nanoparticles [57]. Similarly, Ahmed et al. showed that natural biological variability in plant-mediated synthesis often results in limited control over particle uniformity and poor reproducibility between batches, which continues to hinder large-scale and reliable production of green-synthesized nanoparticles [58].

#### 4.3.2. Microfluidic Synthesis

A viable substitute for other traditional techniques for producing AuNPs is microfluidic synthesis, which uses microfluidic devices to produce stable nanoparticles with regulated size and shape distributions. Compactness, controllability, process fineness, stability, and a little reaction amount are among the advantageous properties revealed by the microfluidics technique. The manufacturing of nanoparticles with unique homogeneous sizes, forms, and morphologies used in a variety of bioapplications, including medication delivery, healthcare, and biosciences—has recently been made possible by the integration of fluid mechanics with microfluidics technology [8]. Yang et al. investigated the synthesis of peptide-mediated AuNPs using droplet microfluidics, demonstrating the possibility of AuNPs with sizes ranging from hundreds of nanometers to the micron scale. The study’s findings highlighted how microfluidics synthesis can progress AuNP manufacturing for novel biomedical and technological uses [59].

Although microfluidic synthesis offers excellent control over particle size and improved reproducibility, it still faces several practical limitations. One of the main technical problems is channel clogging, which can occur as nanoparticles gradually accumulate inside the microchannels, particularly at higher concentrations or during extended operation. Moreover, while microfluidic systems perform very well at the laboratory scale, scaling up continuous production for industrial use remains challenging. This is mainly because increasing throughput requires the parallel operation of many channels, which adds complexity and cost to the system. Together, these issues currently restrict the widespread application of microfluidic synthesis for large-scale manufacturing of AuNPs [60].

#### 4.3.3. Machine Learning Methods

Effective AuNPs and other nanoparticles can be developed using machine learning, which has promise for use in cutting-edge biomedical applications such pharmaceutics, chemical sensing, thermoelectrics, and medical diagnostics. When ML is used in AuNPs, several features are created using the algorithms [9]. In order to learn the mathematical correlations between experimental parameters like concentration, temperature, and reactant type, as well as nanoparticle attributes like size and shape, the synthesis process entails prediction problems. In order to predict the cellular uptake of AuNPs, Bilgi et al. trained machine learning models using 2077 data points for 193 nanoparticles, demonstrating how ML can assist in finding important parameters that influence AuNP uptake. The results indicated that the most crucial elements for AuNP cellular uptake were particle size, zeta potential, concentration, and exposure duration; hence, ML concentrates on creating sophisticated AuNPs that can support novel medical applications [61]. To create the synthesis and manufacturing of AuNPs using AI and advanced medical technologies, more research is necessary. Recent developments in self-driving lab systems enable real-time automated control of nanoparticle synthesis, increasing the accuracy and consistency of gold nanoparticle production and expanding its applications, according to a study by Wu et al. (2025) [62].

Even though AI and machine learning are very promising in AuNP research, one of the main problems is still the lack of large and well-standardized datasets. Most existing models depend on small datasets collected under different experimental conditions and reported in different ways. Because of this, the predictions are not always reliable, and the wider use of these models is still limited.

When the main synthesis routes are compared quantitatively, clear differences emerge in particle size, morphology, and stability. The Turkevich method typically yields spherical AuNPs with sizes of about 10–30 nm and good stability in aqueous media, whereas the Brust–Schiffrin method produces much smaller particles (approximately 1.5–5 nm) with high long-term stability due to strong thiol surface capping. Seed-mediated growth is mainly used for the preparation of anisotropic structures such as nanorods, where the aspect ratio can be tuned to shift optical absorption into the near-infrared region. Green synthesis generally gives larger and more polydisperse particles (20–100 nm), reflecting the variable composition of biological reducing agents. In contrast, microfluidic synthesis allows tighter control over particle size and batch-to-batch reproducibility, often resulting in narrow size distributions. These quantitative differences directly influence optical behaviour, surface reactivity, and colloidal stability, and therefore strongly affect biomedical performance.

## 5. Functionalization of AuNPs

**Figure 4:**
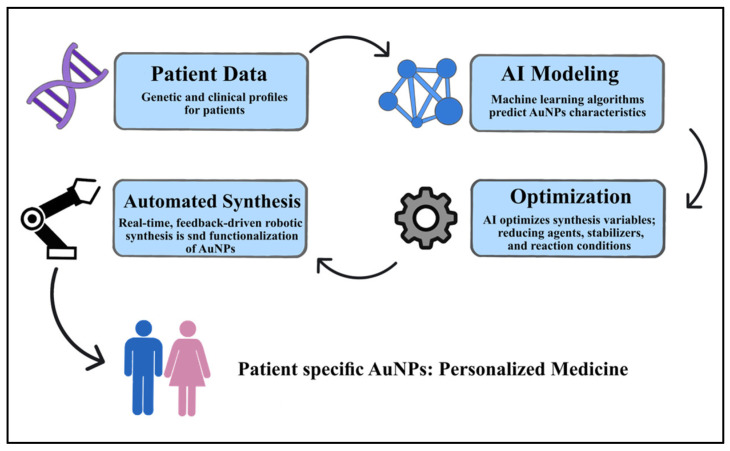
AI-guided modular synthesis of gold nanoparticles (AuNPs).

AuNPs do, however, have several physiochemical characteristics, such as high surface area, tunability, SPR, and strong biocompatibility. Since functionalization enables precise targeting, improved stability, regulated release kinetics, and biocompatibility, it is necessary to bridge the gap between the properties of raw nanoparticles and biomedical applications, thereby establishing AuNPs as important players in biology and medicine [63]. The traditional and sophisticated functionalization techniques are shown in Figure 4.

### 5.1. Conventional Functionalization Methods

The process of directly attaching substances such as medications, proteins, DNA/RNA, or enzymes to the surface of AuNPs is known as ligand functionalization [11]. This method demonstrates how AuNPs can bind thiol and amine groups to allow for alterations for a range of medical uses. Podsiadlo et al. demonstrated that strong sulfur–gold (Au-S) bonds can be used to functionalize AuNPs using 6-Mercaptopurine (6-MP) and its riboside derivatives (6-Mercaptopurine-9-β-D-Ribofuranoside, 6-MPR) [64]. When compared to the drug’s free form, the results demonstrated a significant improvement in the antiproliferative effects of these AuNPs against K-562 leukemia cells, indicating the potential for ligand-based functionalization to increase therapeutic efficacy.

### 5.2. Advanced Functionalization Methods

For biological and medical purposes, AuNPs can be further functionalized by conjugating with different medicines by polymer functionalization. The design and preparation of polymer-functionalized AuNPs have drawn increasing attention in recent years. Therapeutic and biological uses, regulated drug release, and increased biocompatibility and stability are all facilitated by polymer-functionalized AuNPs [3]. The AuNRs–doxorubicin combination was created by Venkatesan et al. [62] by an electrostatic interaction between the negatively charged PSS-AuNR surface (poly(sodium 4-styrenesulfonate)) and the amine group (−NH_2_) of doxorubicin.

Compared to free doxorubicin, these AuNR–doxorubicin conjugates demonstrated improved biological stability and greater therapeutic efficacy. This demonstrates the impact of sophisticated AuNP functionalization techniques on biomedical applications.

Ligand functionalisation is generally preferred when specific biological recognition or active targeting is required, such as in targeted cancer therapy, diagnostic imaging, and biosensing, where antibodies, peptides, aptamers, or small molecules enable receptor-mediated uptake and high binding selectivity [46]. In contrast, polymer functionalisation is favoured for applications that require prolonged circulation, enhanced colloidal stability, and reduced protein corona formation, particularly in systemic drug delivery and imaging, where polymers such as PEG provide steric stabilisation in vivo [65]. Therefore, ligand-coated AuNPs are best suited for precision targeting and sensing applications, whereas polymer-coated AuNPs are more appropriate for long-circulating therapeutic and diagnostic platforms.

## 6. Biomedical Applications of AuNPs

Before discussing specific biomedical applications, it is important to consider the pharmacokinetics, biodistribution, and clearance behaviour of AuNPs in vivo, as these factors critically determine their safety and therapeutic performance. Gold nanoparticles show size- and surface-dependent pharmacokinetics that directly affect their in vivo distribution and clearance. After systemic administration, AuNPs mainly accumulate in the liver and spleen due to uptake by the mononuclear phagocyte system, while ultrasmall particles (<10 nm) may undergo partial renal clearance [22,23]. Surface functionalisation, especially PEGylation, prolongs blood circulation by reducing protein adsorption and immune clearance, thereby improving tumor accumulation through the enhanced permeability and retention (EPR) effect [40].

AuNPs’ distinct properties, variety of synthesis techniques, and sophisticated biological functionalization strategies demonstrate their revolutionary importance in medicinal applications. Gene therapy, targeted medication administration, imaging, and multimodal treatment all benefit greatly from their size, shape, and optical characteristics. A comparison of the uses of gold nanoparticles in biomedicine is shown in Table 2.

### 6.1. Conventional Biomedical Applications

#### 6.1.1. Molecular Delivery System

The potential of AuNPs as delivery vehicles for other compounds, such as medications, DNA, and proteins, has drawn a lot of interest [15]. Chemotherapy has drawbacks, such as the drug’s widespread dispersion throughout the body, which can lead to systemic toxicity and decreased effectiveness against the intended tumor [72]. It is feasible to provide efficient targeted transport and get over the body’s biochemical barriers by employing AuNPs as drug delivery systems (DDS) that target particular cells, tissues, or organs [73]. In addition to their function in delivering diverse pharmaceuticals by binding through chemical or hysical conjugation systems, AuNPs can also deliver other drugs, such as anticancer agents [74]. Glutathione-stabilized gold nanoparticles (GSH-AuNPs) modified with doxorubicin (DOX) by non-covalent conjugation have been explored by Wójcik et al. for possible anticancer drug delivery. Results from the MTT (3-(4,5-dimethylthiazol-2-yl)-2,5-diphenyltetrazolium bromide) assay demonstrated that GSH-AuNPs were substantially more active against feline fibrosarcoma cell lines than unmodified AuNPs [75]. This demonstrates how crucial AuNPs are as DDS for enhancing targeted transport and chemotherapeutic efficacy. Because of their stability, optical characteristics, and biocompatibility, AuNPs have been essential in drug administration and cancer chemotherapy, supporting this idea. In order to provide controlled release and targeted distribution of medications, construction techniques have been created [76].

AuNPs can be used in gene therapy, which targets and treats diseases by employing exogenous DNA or RNA, in addition to drug delivery. Covalent and non-covalent bonding are two methods whereby metallic nanoparticles, and more especially AuNPs, might be joined to oligonucleotides [77]. One example of a highly targeted technique for gene delivery applications is the ability of covalent AuNPs to activate genes in peripheral blood mononuclear cells [77]. In order to target hepatocytes and deliver the pCMV-Luc DNA (pDNA) to liver cancer cells in vitro, Zenze et al. (2024) [66] created receptor-targeting AuNPs coated with chitosan (CS), polyethyleneglycol (PEG), and lactonic acid (LA) (PEG–CS–LA–AuNPs). Comparing targeted AuNPs to non-targeted formulations, the results revealed a noteworthy five-fold increase in luciferase gene expression [66]. The fact that AuNPs exhibit great biocompatibility, increased transgene activity, and the possibility for liver-related gene therapy highlights their function in gene delivery in many cancer disorders.

AuNPs’ use in several therapeutic applications, such as the control of diabetes, is enhanced by their significant role as carriers for protein delivery [15]. Joshi et al.’s study demonstrated how insulin developed when it was covalently linked to AuNPs (Au-Insulin) [67].

When compared to insulin-bound via hydrogen bonds with amino acid-modified AuNPs for the treatment of diabetes, this connection demonstrated noticeably greater action. The results demonstrated how well AuNPs carry proteins, which improves the efficacy of therapeutic applications for common illnesses like diabetes.

#### 6.1.2. Basic Diagnostics and Imaging

AuNPs improve contrast and allow for precise tissue viewing, which leads to several improvements in therapeutic and diagnostic imaging procedures such as X-CT [7]. AuNPs are becoming more and more popular in imaging as an X-ray contrast agent because they can absorb ionizing radiation, improve the quality of X-ray absorption, and use optical processes like SPR to turn light into heat energy [78]. Because of their distinct X-ray absorption coefficient, iodinated molecules are typically employed as a contrasting agent; nevertheless, their short imaging periods and quick renal clearance present serious drawbacks [76,79]. However, AuNPs are a very good substitute as a contrast agent for X-ray imaging because of their distinct optical and electrical characteristics, high electron density, greater atomic number of Au, and even higher absorption coefficient for X-rays [43]. To investigate the possibility of using imaging to track and quantify protease activity in vivo, Liu et al., for example, created 30–40 nm gold nanocages (AuNCs) as a component of an activatable probe [80]. With gold nanoparticles’ valuable features and benefits over other materials, this study offers insights into novel potential for diagnostic imaging. Additionally, Ibrahim et al. (2024), [68] discovered that spectral photon-counting CT allows for accurate gold nanoparticle discrimination and quantification in imaging experiments, and that gold nanoparticles produce greater X-ray attenuation than iodine [68].

#### 6.1.3. Biosensing Applications

Because of its high surface-to-volume ratio, electrical conductivity, and capacity to enhance electrochemical signals, AuNPs have also been connected to biosensing. These applications in nanoparticle-mediated signal amplification were established by Liu et al. (2025), [69]. who demonstrated the one-step detection of several breast cancer indicators in blood. Additionally, AuNPs-based biosensors are being used increasingly often in infectious illness and cancer diagnostics, demonstrating more dependable and effective results than traditional techniques [81].

### 6.2. Advanced Biomedical Applications

#### 6.2.1. Hybrid AuNPs: Theranostics

By integrating medicines and diagnostics into a single system, theranostics makes diagnosis and treatment easier and tracks the effectiveness of treatments [82].

As previously stated, AuNPs’ special qualities, such as their increased biocompatibility, tuneability, and SPR, make them appropriate for use in cancer theranostics [83]. Methoxy poly(ethylene glycol)–poly(ε-caprolactone)–methotrexate@Au mPEG-PCL-MTX@Au is a hybrid nanoparticle system that combines MTX with a polymeric prodrug (mPEG-PCL) and AuNPs for simultaneous chemotherapy and radiation, according to a study by Hooshyar et al. [81]. The results demonstrated how this hybrid nanoparticle enhanced cancer therapy and stimulated the generation of ROS in cancer cells, underscoring the potential for future advanced medical uses. A composite of mesoporous silica-coated gold nanorods loaded with doxorubicin (AuNR@S-MCM-41-DOX) was also created by Deinavizadeh et al. (2024) [70] that combines doxorubicin with photothermal activity. In comparison to signal treatment, this combination demonstrated dual-responsive drug release behaviors that were pH/NIR and demonstrated a markedly better cytotoxicity against A549 lung cancer cells [70]. Building on these experimental theranostic approaches, Zhuang et al. showed that gold nanoparticles can be engineered as multifunctional theranostic systems that enable tumor-targeted drug delivery, near-infrared photothermal ablation, and simultaneous imaging guidance within a single platform, while surface functionalisation was used to improve tumor penetration and reduce treatment resistance [84].

#### 6.2.2. Advanced Gene Editing

CRISPR/Cas9 technology has emerged as a very effective cell gene editing tool in recent years. However, there was a major problem with the system’s distribution, which limited the use of in vivo gene therapy. In addition to their important function as gene carriers, AuNPs are essential for gene editing technologies [84]. In a work by Konstantinidou et al. (2024) [71], the Cas9 enzyme was conjugated to 12 nm AuNPs through affinity binding between the protein’s 6x His-tag and the NTA-Ni^2^LJ groups on the nanoparticles. This nanoformulation showed gene editing efficacy on par with traditional methods and allowed spontaneous cellular uptake. In a different example, Shahbazi et al. (2019) [85] highlighted the usage of AuNPs core with the citrate reduction approach and created a CRISPR nanoformulation employing colloidal AuNPs (AuNPs/CRISPR), which facilitate homology-directed repair by having nuclease and guide RNA on their surface. The result was effective gene editing. Additionally, they showed that whole CRISPR sequences may be delivered to human blood stem and progenitor cells without causing harm [85].

Sometimes, the CRISPR system can cut DNA at unintended sites, which may lead to unwanted mutations [86]. Studies have also shown that keeping the Cas9 enzyme active for a long time increases this risk [87]. For this reason, CRISPR delivery systems based on AuNPs must be carefully designed to work for a short time and in a controlled way [71]. In addition, proper guide RNA design, dose control, and long-term animal studies are necessary before these systems can be safely used in humans [85].

In general, AuNPs’ special qualities led to their promise in a variety of biomedical applications. Table 3 offers a thorough synopsis that connects these characteristics to research on biomedical applications.

## 7. Future Perspectives for AuNPs

### 7.1. Traditional Translational Pathways for AuNPs

Although gold nanoparticles show great promise in research, translating them into real clinical use is still challenging. Large-scale production with consistent quality remains difficult, and regulatory approval requires long-term data on toxicity, biodistribution, and clearance, which is still limited for many formulations. In addition, differences in synthesis methods and surface coatings make standardization difficult, slowing down clinical translation despite strong experimental results.

### 7.2. AI-Guided Modular AuNP Synthesis for Personalized Medicine

We have covered the function of AI in functionalizing and preparing AuNPs for new manufacturing techniques in this review. Developing AI-guided modular systems for AuNP synthesis that are tailored to certain therapeutic requirements is the goal. To improve synthesis settings, these platforms might take advantage of patient-specific data, like genetic profiles.

This individualized method would enable the creation of nanoparticles with unique chemical and physical characteristics for every patient. By developing a just-in-time production paradigm, this approach might aid in overcoming the drawbacks of traditional nanoparticles for applications in personalized nanomedicine. The procedure of developing AuNPs for use in biological applications is shown in Figure 4.

### 7.3. Self-Learning AuNP-Based Theranostic Devices

AI algorithms used with theranostic devices would combine the therapeutic and diagnostic properties of AuNPs and offer real-time functionality. These systems, which can be created by evaluating data obtained from biosensors, would allow for on-demand adjustments to therapy parameters, such as medication release rates or intensity, to accommodate the state of each patient and the course of the disease. This could enhance the accuracy and effectiveness of different cancer treatments while minimizing harm to healthy tissues, which could be particularly helpful for aggressive or quickly changing tumors.

## 8. Conclusions

This review aims to provide an up-to-date overview and comparison between classical and innovative solutions to the challenges addressed in the field of the synthesis, functionalization of gold nanoparticles (AuNPs) and their biomedical applications, (as shown in Table 4). However, although chemical reduction and seed-mediated growth were the most common techniques used in AuNP preparation, these less-inclusive approaches lack the sophistication and capabilities of newer techniques such as green synthesis, microfluidics and artificial intelligence-enabled optimization can bring in terms of scalability, accuracy, and environmental sustainability. Functionalization has also evolved from simple ligand tagging to more complex polymer and hybrid systems, improving stability and targeting. Furthermore, the application of AuNPs has developed from a simple drug carriers and imaging agents to a multi-functional platforms for theranostics, to CRISPR gene editing, and finally to adaptive therapies. This comparative study demonstrated the potential for AuNPs to resolve current limitations and broaden their application by addressing novel materials for personalized medicine and advanced biomedical solutions.

## Figures and Tables

**Table 1:** Synthesis Methods of Gold Nanoparticles.

Method	Description	Key Features
Turkevich Method	Classical chemical reduction using trisodium citrate as reducing and stabilising agent for Au^3+^ ions.	Produces hydrophilic spherical AuNPs (10–100 nm) with good monodispersity.
Brust–Schiffrin Method	Two-phase organic reduction employing tetraoctylammonium bromide and sodium borohydride.	Generates small (1.5–5.2 nm), stable AuNPs transferable to organic media.
Seed-Mediated Growth	Growth of small gold seeds to form nanorods and other anisotropic structures.	Allows precise control of shape and aspect ratio.
Green Synthesis	Uses plant or microbial extracts as natural reducing and stabilising agents.	Eco-friendly, biocompatible route suitable for biomedical use.
Microfluidic Synthesis	Employs continuous-flow microreactor systems for controlled nucleation and growth.	Produces uniform particles with reproducible size.
Machine-Learning-Assisted Synthesis	Optimises reaction parameters such as temperature and concentration through data-driven algorithms.	Provides reproducibility and morphology control.

**Table 2:** Biomedical Applications of Gold Nanoparticles.

Application	Example	Mechanism	Type of Study	Reference
Drug Delivery	GSH-stabilised AuNPs modified with doxorubicin (DOX) through electrostatic interaction between the amine group (−NH_2_) of DOX and the negatively charged PSS–AuNR surface.	These AuNR–DOX conjugates showed enhanced biological stability and higher therapeutic efficacy compared with free DOX.	Polymer-functionalised AuNR–DOX conjugates.	Venkatesan et al. (2013) [62]
Gene Therapy	PEG–CS–LA–AuNPs used for the delivery of plasmid DNA (pCMV-Luc) to hepatocytes.	The PEG–CS–LA–AuNPs achieved a five-fold increase in gene expression.	PEG–CS–LA–AuNP/pDNA complexes.	Zenze et al. (2024) [66]
Protein Delivery	Insulin conjugated to AuNPs via covalent binding.	The insulin–AuNP conjugates provided improved stability and sustained release.	Insulin-bound AuNPs.	Joshi et al. (2006) [67]
Imaging/ Diagnostics	Au nanocages (30–40 nm) used as X-ray contrast agents.	The Au nanocages produced higher attenuation and clear tumour delineation.	Au nanocages (30–40 nm).	Ibrahim et al. (2024) [68]
Biosensing	AuNP-based immunosensors applied for the detection of specific biomarkers.	AuNPs enhance detection through optical and electrochemical signals.	AuNP-based immunosensors.	Liu et al. (2025) [69]
Theranostics/Photothermal Therapy	mPEG–PCL–MTX@Au and AuNR@S–MCM–41–DOX systems responsive to pH and NIR irradiation.	These systems demonstrated pH/NIR-responsive drug release and enhanced cytotoxicity.	mPEG–PCL–MTX@Au and AuNR@S–MCM–41–DOX hybrids.	Deinavizadeh et al. (2024) [70]
Gene Editing	Cas9–AuNP conjugates used for delivering CRISPR components into target cells.	These conjugates provided efficient and precise genome modification with low toxicity.	Cas9–AuNP conjugates.	Konstantinidou et al. (2024) [71]

**Table 3:** Properties and Biomedical Applications of AuNPs with Studies.

Property	Description	Applications
Surface Plasmon Resonance (SPR)	Unique optical property that enhances light absorption and scattering.	Biosensing and imaging.
Tunable Size and Shape	Ability to control shape and size for specific biomedical functions.	Molecular imaging, targeted therapy, and CRISPR/Cas9-assisted therapies.
Functionalization Potential	Enables attachment of ligands, polymers, and biomolecules for targeted delivery.	Targeted drug delivery, gene therapy, and immune modulation.
Biocompatibility	Low cytotoxicity and compatibility with biological systems.	Theranostics and basic drug delivery systems.
Photothermal Conversion	Efficient conversion of light energy into heat for therapeutic use.	Cancer therapy and photothermal ablation.

**Table 4:** Comparative Summary of Conventional and Advanced Approaches for Gold Nanoparticles.

Aspect	Conventional Approaches	Advanced Approaches
Synthesis Methods	Chemical and physical methods are considered essential and the base for AuNP production, including reduction of gold ions using different reducing and stabilizing agents.	Green eco-friendly systems using plant or microbial extracts, microfluidic synthesis that provides controlled size and morphology, and machine-learning (ML) integrated synthesis for precision and reproducibility.
Functionalization Techniques	Ligand functionalization through thiol and amine groups for the direct attachment of drugs, proteins, or nucleic acids to the surface of AuNPs.	Polymer and hybrid functionalization using PEGylation or smart polymer coatings that improve biocompatibility, stability, and controlled release for targeted delivery.
Applications	Conventional uses include CT and X-ray imaging where AuNPs act as contrast agents, and basic molecular or drug-delivery systems for chemotherapy.	Advanced biomedical applications involve photothermal and photodynamic cancer therapy, hybrid AuNP-based theranostic systems, and CRISPR/Cas9-assisted gene editing for precision medicine.

## Data Availability

No new data were created or analyzed in this study. Data sharing is not applicable to this article.

## Abbreviations

The following abbreviations are used in this manuscript:

Abbreviation	Full Term
6-MP	6-Mercaptopurine
6-MPR	6-Mercaptopurine-9-β-D-Ribofuranoside
AI	Artificial Intelligence
Au	Gold
AuNP(s)	Gold Nanoparticle(s)
AuNCs	Gold Nanocages
AuNR(s)	Gold Nanorod(s)
AuNR@S–MCM–41–DOX	Mesoporous-silica-coated gold nanorods loaded with doxorubicin
BBA	Biochimica et Biophysica Acta
Cas9	CRISPR-Associated Protein 9
CRISPR	Clustered Regularly Interspaced Short Palindromic Repeats
CT	Computed Tomography
CTAB	Cetyltrimethylammonium Bromide
DDS	Drug Delivery System
DNA	Deoxyribonucleic Acid
DOX	Doxorubicin
EG	Ethylene Glycol
FDA	Food and Drug Administration
FRET	Förster Resonance Energy Transfer
GSH	Glutathione
HAuCl_4_	Hydrogen Tetrachloroaurate
LA	Lactonic Acid
LiAlH_4_	Lithium Aluminium Hydride
LSPR	Localized Surface Plasmon Resonance
MCF-7	Michigan Cancer Foundation-7 (human breast cancer cell line)
ML	Machine Learning
mPEG–PCL	Methoxy Poly(ethylene glycol)–Poly(ε-caprolactone)
MTX	Methotrexate
MTT	3-(4,5-Dimethylthiazol-2-yl)-2,5-Diphenyltetrazolium Bromide
NaBH_4_	Sodium Borohydride
NaOH	Sodium Hydroxide
NIR	Near-Infrared
NP(s)	Nanoparticle(s)
pDNA	Plasmid Deoxyribonucleic Acid
PEG	Polyethylene Glycol
PEG–CS–LA–AuNPs	PEG–Chitosan–Lactonic Acid–Gold Nanoparticles
PLAL	Pulsed Laser Ablation in Liquid
PSS	Poly(sodium 4-styrenesulfonate)
PDT	Photodynamic Therapy
PTT	Photothermal Therapy
RNA	Ribonucleic Acid
ROS	Reactive Oxygen Species
SDS	Sodium Dodecyl Sulfate
SERS	Surface-Enhanced Raman Spectroscopy
SPR	Surface Plasmon Resonance
TNF-α	Tumor Necrosis Factor-Alpha
UV	Ultraviolet
X-CT	X-ray Computed Tomography
